# Active cooling device: A flexible, lab-scale experimental unit to develop spatio-temporal temperature control strategies

**DOI:** 10.1016/j.ohx.2026.e00754

**Published:** 2026-02-28

**Authors:** Victor Oliveira Ferreira, Wiebke Mainville, Vincent Raymond, Jean-Michel Lamarre, Antoine Hamel, Mikael Vaillant, Moncef Chioua, Bruno Blais

**Affiliations:** a Chemical engineering High-performance Analysis, Optimization and Simulation (CHAOS), Department of Chemical Engineering, Polytechnique Montréal, PO Box 6079, Stn Centre-Ville, Montréal, QC, Canada, H3C 3A7; b National Research Council Canada, Boucherville, Québec, Canada; c Department of Chemical Engineering, Polytechnique Montréal, PO Box 6079, Stn Centre-Ville, Montréal, QC, Canada, H3C 3A7

**Keywords:** Thermal management, Active cooling, Process control, Raspberry Pi, Impinging jets

## Abstract

This work proposes an experimental unit that realizes a multi-input, multi-output manifold thermal management technology. The proposed setup is designed for experiments aimed at controlling spatiotemporal temperature distribution. Temperature control is achieved by impinging coolant fluid jets, leveraging a manifold of channels targeted to the surface. The direction of the fluid is controlled by shifting the role of channels between inputs, outputs, or closed states. Files associated with this work include Computer-Aided Design (CAD) STEP files, Gerber files to manufacture a custom Printed Circuit Board (PCB), and a Graphical User Interface (GUI) written in Python. A step-by-step guide to assembling the experimental setup is provided, alongside instructions to interact with the setup through the GUI for real-time tracking. Validation experiments characterize the dynamic performance of the system, demonstrating a temperature reduction of 6 °C in response to a 54 L min^−1^ step change in flow rate, with a settling time of 400 s. Setpoint tracking capability is demonstrated through a representative proportional–integral (PI) control experiment, which consistently reaches the target temperature with high reproducibility across repeated trials. Disturbance rejection performance is further validated by maintaining a 100 °C temperature setpoint under spatially varying heat loads using PI control. With a total component cost of approximately $14,000 USD, the active cooling device presents a safe, flexible, and complete design, allowing for lab-scale assessment of the performance of custom temperature control strategies using enclosed impinging jets.

## Highlights

• Modular framework for spatio-temporal thermal management validation.
• Reproducible platform with open-source CAD, PCB, and Python GUI.
• Safe lab-scale testing of adaptive multi-input control strategies.
• Quantified thermal response and disturbance rejection benchmarks.
• Integrated hardware–software stack for reconfigurable flow control.

## Graphical abstract

**Figure:**
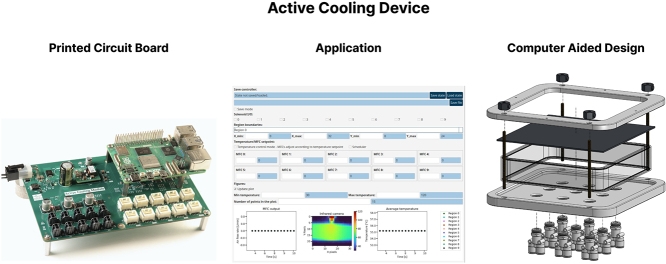

## Specifications table

**Table:** 

**Hardware name**	Active cooling experimental setup

**Subject area**	•Manufacturing and process engineering • Process control • Thermal management

**Hardware type**	•Thermal control device • Lab-scale twin

**Closest commercial analog**	No commercial analog is available.

**Software open-source license**	BSD 3-Clause License

**Hardware open-source license**	Creative Commons Attribution 4.0 International

**Cost of hardware**	Approximately 14,000.00 USD, including only the price of off-the-shelf material. Costs do not include manufacturing and design.

**Source file repository**	https://doi.org/10.5281/zenodo.15644038

## 1 Hardware in context

Thermal management is among the most important and sensitive operations in several industries. From asserting part quality upon manufacturing to optimizing the performance of electronic components and batteries, cooling and heating systems are key components in processes involving high thermal loads and chemical reactions. For example, in die casting or pressure molding, the mechanical properties of the manufactured parts are highly dependent on the cooling rate [1]. When this cooling rate is poorly controlled, parts can suffer uncontrolled shrinkage and might require additional heat treatment to achieve the desired properties. As most parts present irregular geometry and uneven mass distribution, accounting for the shape of the part while designing the cooling system of the mold is challenging. One way to achieve this is by using conformal cooling channels, an effective yet case-specific method that requires advanced manufacturing technology such as 3D printing [2].

The electrification of the automotive sector presents other significant thermal challenges. High-energy-density storage devices, such as Lithium-ion batteries (LIBs), are essential for electric vehicles but generate significant heat per cycle [3]. Effective thermal management is required to prevent impedance increases leading to premature failure caused by overheating and capacity loss caused by low temperatures. Consequently, the lack of adaptive thermal control systems limits the application of technologies dependent on high-density energy storage. Recently, several studies have reported methods of measuring [4], [5], [6], predicting [7], and controlling such temperature [8]; highlighting the need for further development of a robust thermal management technology suitable for high-energy-density batteries.

For these reasons, developing an adaptive temperature control device is key for sensitive industries. In this context, Lamarre and Raymond [9] proposed a flexible active cooling system, consisting of a chamber with a manifold of inlet/outlet pipes applied to control the flow of a cooling and heating fluid. The active temperature control device is designed to control, in time and space, the temperature profile across a surface. This novel technology can be used to impose higher cooling rates in higher temperature regions, ensure even temperature distribution on a surface regardless of the heat load, or even serve as a local heat source for parts with uneven shapes or regions where losses are higher.

A device using a similar principle has been reported by Hopmann et al. [10] and applied for polymer pressure molding. Nevertheless, their device differs from the one proposed by Lamarre and Raymond [9] in terms of heat-transfer mechanism: in the former, the injected polymer is heated by conduction using a heating element and cooled by gas expansion, whereas in the latter, cooling is achieved by forced convection via jet impingement. The enclosed impinging jets can interact more or less depending on the operational parameters applied (such as fluid temperature, jet flow rate, and the ratio between the diameter of the jet and its distance to the surface [11], [12], [13]), which may lead to non-trivial interaction between controllers actuating in different regions.

While the novel active cooling device is promising, the control strategy for specific targeted applications remains unclear. Additionally, testing strategies in production environments is resource-intensive and presents significant safety risks, particularly in high-energy density applications. As such, the industrial adoption of this technology is currently limited by the lack of a testing platform for control strategies.

On the other hand, the integration of embedded systems (such as Arduino boards, Raspberry Pi, and others) with scientific instrumentation has been popularized, enabling reliable, portable, low-cost experimental setups [14]. As an example in the field of flow control, Niketa et al. [15] reports an experimental platform for automated Chemical Vapor Deposition in which flow valves and Mass Flow Controllers (MFCs) are controlled by a Raspberry Pi (RPi). Following a similar concept, this work realizes the active cooling device proposed by Lamarre and Raymond [9]. It presents an open-access version of the device suitable for research trials at a laboratory scale. The primary objective of this work is to provide an accessible transition from a theoretical concept to a fully realized, validated, and open-source experimental platform. This hardware provides a low-cost, safe, flexible, and easily reproducible setup, specifically designed to assess the performance of temperature control strategies involving enclosed impinging jets.

The contributions of this study include:

• Hardware realization, with a thorough description and open-source release of the physical setup used for system characterization.
• Electronic integration, with the design of a custom Printed Circuit Board (PCB) that integrates a Raspberry Pi with up to 10 MFCs, 10 solenoid valves, and an infrared camera into a single manageable unit.
• Modular software, including a complete backend and Graphical User Interface (GUI) developed in Python with PySide6 [16], [17].

The combined hardware, electronic and software allows researchers to implement, test and validate custom control strategies on the active cooling device. Finally, this work briefly illustrates some potential use of the setup through showcase experiments that emulates thermal disturbances. By enabling the safe validation of control strategies – such as the disturbance rejection and setpoint tracking – this device serves as a bridge between the design proposed Lamarre and Raymond [9] and its practical application in the targeted sectors like battery storage and precision manufacturing.

## 2 Hardware description

**Fig. 1:**
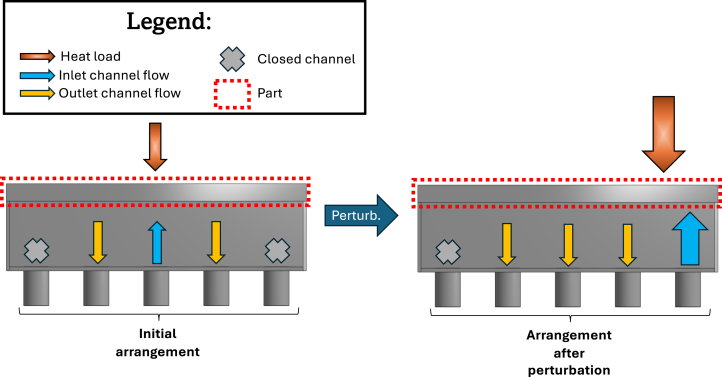
Illustration of the perturbation rejection principle of the active cooling device. On the left, the system is at steady-state using the initial arrangement. When the heat load is shifted to the right and increased in magnitude, the system reacts, shifting the arrangement and adjusting the inlet flow rate accordingly.

The active cooling device uses enclosed impinging jets to control the temperature profile of a metallic plate. The principle behind the temperature control is illustrated in Fig. 1. The part (highlighted by the red-dotted rectangle) is exposed to a localized heat load, represented by the orange arrow pointing to the plate. In response to a perturbation, the desired temperature profile of the part is regained by controlling the inlet and outlet flow within the chamber below the part, represented by the arrows and “x” symbols within it. Each of the five channels can act as an inlet, an outlet, or be closed. The same principle can be applied to any number of jets.

In the process in Fig. 1, the target temperature profile is achieved by injecting fluid by the central channel (blue arrow pointing up to the plate), opening its neighbors to be outlets (yellow arrows pointing down from the plate), and closing the channels at the extremities (represented by “x” symbols). After a perturbation, the device should react by changing the inlet/outlet/closed channel arrangement and the inlet flow rate (represented by the size of the arrow) in order to maintain the target temperature profile.

**Fig. 2:**
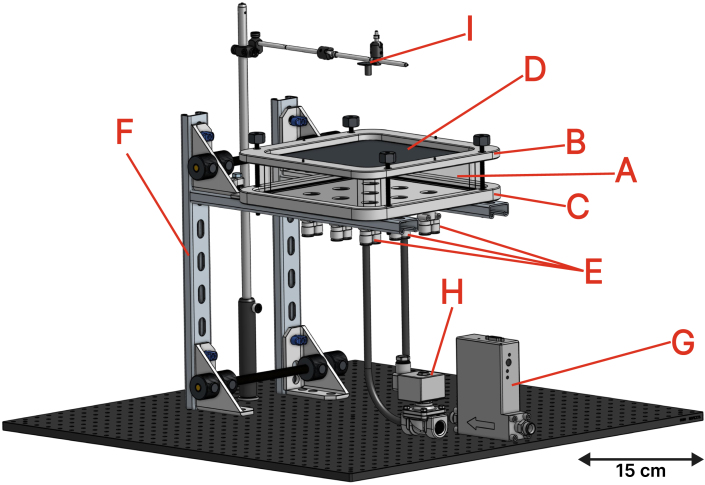
Computer-aided design (CAD) representation of the experimental setup. The enclosed chamber (A) is sealed between the upper part (B) and lower part (C) via threaded rods, with the metal plate (D) positioned between (A) and (B) and rubber o-rings preventing leakage. Y-shaped tube fittings (wye, E) connect the bottom of the chamber to a Mass Flow Controller (MFC, G) on one side and a solenoid valve (H) on the other; each wye has its own MFC–solenoid valve pair, though only one of each is shown for clarity. The chamber is supported by the chamber mount (F), fixed to an optical table. The infrared thermal camera (I) is positioned above the setup to measure the surface temperature distribution of the metal plate.

Fig. 2 shows an isometric illustration of the experimental setup. The device is divided into three units: the heat source, the chamber, and the control hardware.

• *Heat source* - The heat source can vary with the targeted application. In the present work, we use a commercially available heat gun (available on McMaster-Carr under the serial number 3433K56) with air temperature control. The heat gun was chosen for its portability, ability to provide a consistent localized heat load, and for offering a clear line of sight for the infrared camera. The setup can be adapted to a wide range of heat sources, as long as a surface temperature distribution measurement can be obtained.
• *Chamber* - The enclosed chamber (A) is sealed by compressing it between the upper part (B) and the lower (C) using threaded rods. The metal plate (D) is positioned between the upper part (B) and the enclosed chamber (A), while rubber o-rings on each side of the chamber (A) are used to prevent leakage. Y-shaped tube fittings (wye, E) are connected to the bottom of the chamber. Each of those wyes has the same configuration: one side connects the chamber to the Mass Flow Controller (MFC, G), connected to the compressed air line, while the other end is connected to a solenoid valve (H), which opens to ambient air. Fig. 2 shows only one MFC and one solenoid valve, but each wye is connected to its own pair. These connections enable various jet arrangements, as described in the following subsection. Finally, the enclosed chamber is supported by the chamber mount (F), mounted to an optical table.
• *Control hardware* - Comprises the sensors (MFC monitor and IR camera), actuators (MFC control valves and solenoid valves), the custom Printed Circuit Board (PCB), and a Raspberry Pi (RPi) [18]. The RPi communicates with the hardware parts, collecting measurements and acting on the jets according to the implemented algorithm. Details on components are provided in the following subsections.

In the following, we explain the operation of the equipment and the function of each unit. Furthermore, details about the rationale behind the design choices and their associated limitations are provided.

### 2.1 Jet flow rate and arrangements

The temperature control is achieved by manipulating two variables, namely jet arrangement (or arrangement, for brevity) and flow rate. Each of the orifices at the bottom of the chamber can act as air outlets, air inlets, or be shut. The arrangement of the inlet/outlet/shut orifices determines the distribution of the air flow within the chamber, which is used to manipulate the temperature distribution of the top plate (D). Additionally, the flow rate of the inlet jets can be modulated.

**Fig. 3:**
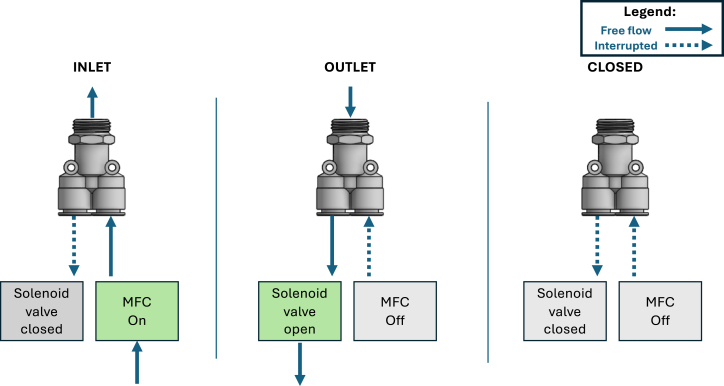
Schematic diagram depicting the jet arrangement manipulation logic.

The arrangement is controlled by the MFCs and the solenoid valves, concomitantly, as shown in Fig. 3. As previously noted, a MFC–solenoid valve pair is connected to a wye flow splitter, which is connected to the orifice of the channel. When the orifice is in a closed state, the solenoid valve controlling the outlet flow is shut, and the MFC flow rate is set to zero. When the orifice is set as an outlet, the solenoid valve downstream of the chamber is open, allowing the passage of air. Lastly, when the orifice is in an inlet state, the solenoid valve is closed, and the MFC controls the inlet flow rate. The equipment operates with at least one orifice as an outlet to prevent pressurization.

The inlet air jet flow rates are controlled by mass flow controllers (MFCs). In this work, we used MFCs model PFCQ531-04-A1C-S from SMC [19]. When operating within the nominal pressure range, MFCs independently measure and control the gas flow rate with a fast settling time (≤0.5 s at 300 kPa) and a large flow rate modulation range (9 to 300 L min^−1^). The RPi sends commands and receives actual flow rate data for each MFC through analog signals. Similar integrations between MFC and RPi have been tested by Niketa et al. [15] and proven to be reliable. However, the present setup extends the concept to significantly higher flow rate magnitudes and a higher number of simultaneously controlled MFCs.

### 2.2 Temperature measurement

We use an MLX90640 infrared camera [20] to measure the temperature across the surface of the plate. The resolution of the camera is 32 × 24, and it is available with field-of-view angles of 110°(MLX90640BAA) or 55°(MLX90640BAB). It communicates with the RPi through the I2C protocol, and the latter stores the temperature information as a vector. The temperature measurements are used for real-time control.

### 2.3 Printed circuit board

**Fig. 4:**
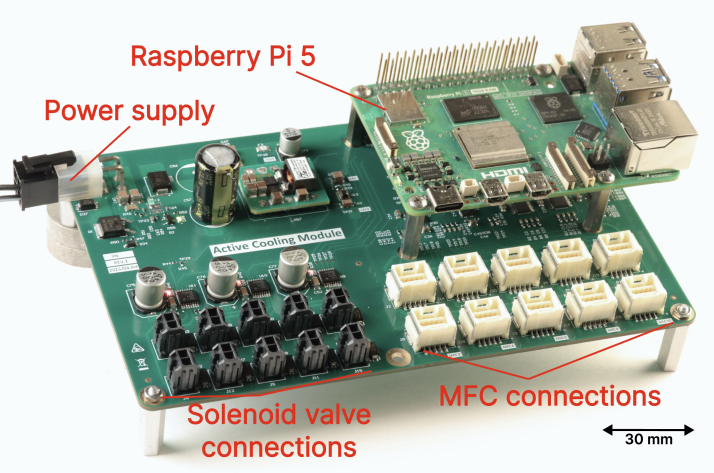
Picture of the custom PCB with mounted Raspberry.

We use a custom Printed Circuit Board (PCB) to connect the electronic components to the sensors, valves, controllers, and the RPi. The custom PCB, depicted in Fig. 4, was designed to feed the entire system using a single 24 V power supply, distributing the correct voltage for each component. The main chips include two Digital-to-Analog converters (DAC, model DAC7578SRGER [21]), two Analog-to-Digital converters (ADC, model TLA2528IRTER [22]), and three motor drivers for solenoid valves (model DRV8806 [23]). DAC and ADC modules communicate with the RPi through the I2C protocol, while the motor driver uses Serial-Peripheral Interface (SPI). A custom Python firmware was developed for communication with the three chips. The firmwares are openly available and can be used with other assemblies using the same chips, independently of the setup. The schematics of the PCB, the code, and all information necessary to reproduce the module are fully available in the Zenodo repository [24] associated with this publication.

### 2.4 Graphical user interface (GUI)

A GUI in Python was created and made available using the PySide6 [16] library to facilitate interaction with the experimental setup. PySide6 was chosen because it is a reliable, high-level, lightweight interface between Python code and the Qt 6 library [17]. Details about the interface are provided in Section 6.

### 2.5 Potential usage of the hardware

The proposed hardware can be used by a broad research community. We highlight a few potential usage:

• **Control strategy benchmarking platform:** Researchers developing novel thermal control algorithms, such as model predictive control (MPC) or advanced decoupling strategies, can use this setup as a testbed to validate and compare controller performance before deployment.
• **Jet impingement heat transfer studies:** The reconfigurable inlet/outlet/closed channel arrangement and the infrared temperature measurements allow researchers studying impingement jet heat transfer to systematically vary jet configurations and quantify their effect on surface temperature distributions, enabling the generation of validation datasets for analytical or computational fluid dynamics studies.
• **Lab-scale digital twin development:** The open-source hardware and software stack, combined with the reproducible and well-characterized thermal response, make this device a practical candidate for developing and validating digital twins or surrogate models of thermal management systems, as demonstrated by Vaillant et al. [25].
• **Embedded systems and instrumentation prototyping:** Researchers working on experiments which require the simultaneous control of multiple MFCs can leverage the open-source electronics design as a reference platform or starting point for their own projects.

## 3 Design files summary

All files described in this section are available on the Zenodo repository associated to this publication (DOI: 10.5281/zenodo.15644038) under Creative Commons Attribution 4.0 International.

**Table:** 

Design filename	File type	Open-source license	Location of the file
Active_cooling_CAD_files.zip	CAD STEP files, including assembly of parts forming the setup.	Creative Commons Attribution 4.0 International	https://zenodo.org/records/15644038

Bill_of_materials.xlsx and Bill_of_materials.csv	Bill of materials in Excel and commas-separated values format.	Creative Commons Attribution 4.0 International	https://zenodo.org/records/15644038

PCB_Design_files.zip	PCB design files, including Gerber, footprint, descriptive Bill of Materials, and all other necessary files to build and assemble the PCB.	Creative Commons Attribution 4.0 International	https://zenodo.org/records/15644038

The CAD step files in the repository can be imported in any STEP compatible CAD design software to recreate the design in Fig. 2. We also included the Bill of Materials, which includes the price (at the time of construction) of each of the main parts used in the assembly. Finally, we also included the PCB design and construction files in the repository, which can be used to reproduce our assembly in Fig. 4 in its totality.

## 4 Bill of materials summary

The bill of materials is available in Appendix and on the online repository [24].

**Fig. 5:**
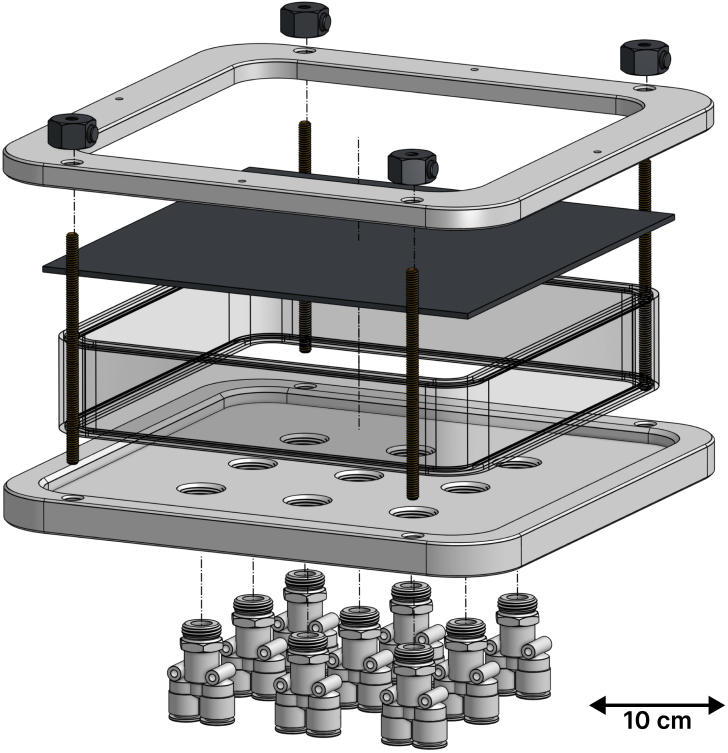
Exploded view of the chamber.

## 5 Build instructions

Most of the setup is composed of off-the-shelf components; therefore, assembling the experimental setup involves connecting the parts according to the design files in the Zenodo repository [24] associated with this publication. The following section explains the process of assembling the setup.

### Chamber mount:

The assembly starts by attaching the chamber mount to an optical table or any similar mounting surface. The base is screwed to the optical table and the strut channel. After this, we screw together the vertical struts and 90° strut channel brackets. Then, we screw the horizontal struts to the strut channel brackets, which hold the chamber. Lastly, we use threaded rods to fix the space between vertical struts and prevent rotation.

### Chamber:

We provide an exploded view of the chamber in Fig. 5. The chamber is composed of the walls, the metallic plate sample, the top component (“hat”), and the base. First, we position the base on the strut. Then, we insert an o-ring into the grooves on either side of the acrylic chamber and place the chamber on top of the base. After this, we place the metallic plate on top of the chamber, followed by the hat. The complete component stack is held together and fixed to the mount with vertical threaded rods as shown in Fig. 5.

### Connection between compressed air line, mfcs, solenoid valve, and chamber:

Regardless of the compressed air source, the MFCs in this work require an upstream pressure between 50 and 800 kPa, with a differential pressure range of 50 and 500 kPa. To fulfill both pressure, differential pressure, and flow rate range requirements, we use 12 mm diameter flexible tubes to connect the MFCs to a compressed air line, supplying air at constant 450 kPa. The connections between tubes and threads are made with a quick-connect adapter. The same applies to the connection between the chamber and the solenoid valves.

### Connection between electronic components:

Electronic components, including MFCs, solenoid valves, and the IR camera, have their unique connector pairs in the PCB. We power the module (PCB, RPi, controllers, and sensors) using a 24 V, 20 A AC/DC converter, connected directly to the PCB.

**Fig. 6:**
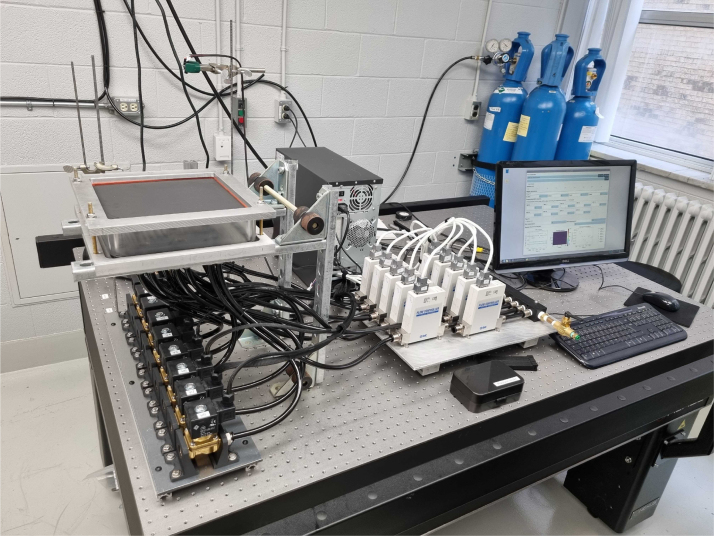
Picture of the setup mounted on the optical table. It includes all parts. Control parts, including the PCB and Raspberry Pi, are inside the computer casing in the image.

The fully assembled system is presented in Fig. 6.

## 6 Operation instructions

**Fig. 7:**
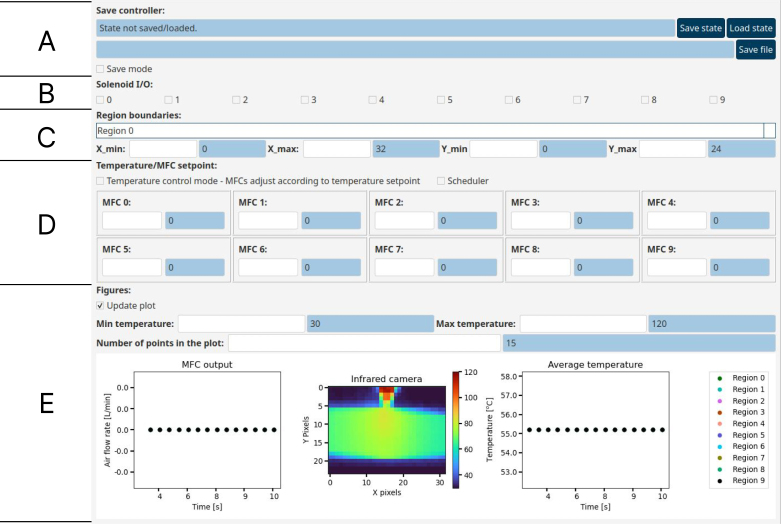
Full Graphical User Interface (GUI).

We operate the equipment through the GUI application. The application is shown in Fig. 7. In this section, the operation of the equipment is explained by describing each individual part of the GUI.

### Region boundaries:

First, we need to assign a sub-domain to each MFC to establish their respective zones of influence. These zones are rectangular, and typically, their centers coincide with the orifice axis (the main axis of each air jet). This is done by inputting the minimum and maximum point coordinates in the x and y axes. By default, when the application starts, all regions cover the entire field of view of the camera, with x going from 0 to 32 and y from 0 to 24.

The number of regions and MFCs is the same. We define the boundary limits of individual sections using the drop-down menu indicating Region 0 in Fig. 7 (C).

### Solenoid I/O:

The solenoid valves control is done using the checkboxes in Fig. 7 (B). Solenoid valves are mapped to their own checkboxes and respond immediately to changes in checkbox state.

### Temperature/MFC setpoint:

The device can take MFC flow rate or target temperature as input. When the Temperature/MFC setpoint checkbox is unchecked (default), the flow rate per MFC is controlled directly by the user. In this mode, each MFC has a framed box allowing the user to manually input the flow rate. Fig. 7 (D) illustrates the use of the interface for the operation with two MFCs. The MFC mode allows us to input the flow rate desired per MFC in L min^−1^. This input becomes the new flow rate set point, constantly displayed in the right text box. When we operate the experimental setup illustrated in Fig. 2, the number of MFCs is 9, and so is the number of framed boxes in this section of the GUI.

**Fig. 8:**
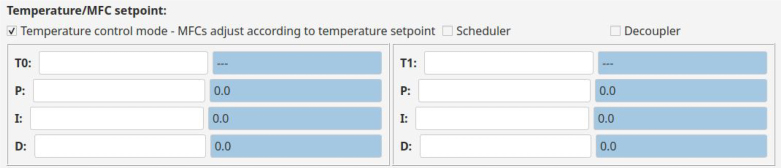
Temperature setpoint and controller gains definition.

As shown in Fig. 8, when the Temperature control mode checkbox is checked, the framed boxes change and the temperature control parameters become available. This mode takes the temperature setpoint and manipulates MFC flow rates to achieve it. Similarly to the MFC mode, when a parameter is set in the left text box, it is displayed in the right text box. The temperature control in the example is done by using a Proportional–Integral–Derivative (PID) control algorithm [26]. The parameters of this controller are the temperature setpoint and the gains of each component (proportional, integral and derivative). The software gathers the average temperature per region and applies the controller output to the MFC flow rate. The control mode also allows for the addition of a standard decoupler, which can be added by checking the checkbox in Fig. 8.

### Scheduler:

When the Scheduler option is checked (unchecked by default), the user is prompted to select a scheduler file. A .csv file, with the first column specifying time and subsequent columns specifying flow rate values for each MFC, allows for automated tests. In Temperature Control Mode, the scheduler uses the file to automate setpoint change tests and the specified values correspond to temperature setpoints. The UI displays the start and end values of the time interval, along with either the flow rate of each MFC or the specified temperature setpoints for each region, depending on the selected mode.

### Figures:

Allows the user to track the experiment information in real-time. It includes three graphics: air flow rate per MFC as a function of time, temperature heat map, and average temperature per region as a function of time. The regions placement and boundaries are illustrated as boxes in the heat map, part (E) of Fig. 7.

The temperature range in the heat map can be modified by changing the value in the Min temperature and Max temperature boxes. We can also change the number of previous points in the time series using the Number of points in the plot setting.

Lastly, we use the Update plot checkbox (checked on, by default) to determine whether the plots are updated or not. By not updating the graphics, we reduce the computational cost of the script execution per iteration of the application, which allows us to reduce the acquisition interval if necessary.

### Save controller:

This section enables the selection of the directory of the data and state files. The state files store information such as the region boundaries and PID gains of the controllers for efficient experiment reproduction. In other words, reproducing an experimental condition only implies loading its state file. Saving and loading the files is as easy as clicking the buttons next to the text line, which contains the path to the current state file (if one exists).

Save file function produces two data files. When in MFC control mode, it contains elapsed time since the last initialization of Save mode (unchecked by default), the flow rates per MFC, the average temperature per region, and the region border points. When in Temperature control mode, it additionally contains the temperature setpoints and the proportional, integral and derivative gains per MFC. The second output file records the time and the temperature per pixel within the heat map.

## 7 Experimental demonstration

We conducted three experiments to demonstrate the performance of the setup: a bump test to assess the response of the system to a step change in mass flow rate, a setpoint tracking experiment to evaluate its ability to regulate the temperature and a disturbance rejection experiment with two PI-controlled inlets. In the latter, a localized heat source was successively applied above each inlet, showing that the heat load can be relocated and that each PI controller primarily regulates the temperature in the zone directly above its corresponding inlet.

These tests characterize the thermal response and control performance of the system. The tests served as an initial assessment of the fundamental thermal and fluid dynamic behavior of the setup while allowing for future control strategy development. All results presented in this section have been processed using a Savitzky–Golay filter with a window length of 10 and a polynomial order of one to reduce noise while preserving flow rate and temperature variations.

### 7.1 Step response characterization

**Fig. 9:**
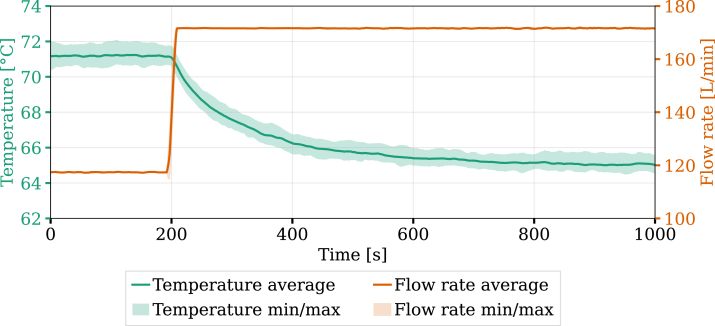
System response to a step change in flow rate.

To quantify the response of the system to a change in mass flow rate, a bump test was performed with a single mass flow controller (MFC). The experiments were repeated four times under identical conditions assess the reproducibility of the methodology using the hardware applied. Fig. 9 shows both the user-imposed step change in flow rate and the resulting temperature response. Results include the average and the range between the minimum and maximum measured values.

The flow rate was increased from 117 to 171 L min^−1^, resulting in a temperature drop from 71 to 65 °C, with a response time of approximately 400 s. A transient phase is observed after the flow rate bump before reaching a new steady-state. The difference between the minimum and maximum temperatures is below 2 °C for the entirety of the interval. Variations in the measured flow rate were negligible. Performing a series of bump tests enables the characterization of the response of the system and provides data for controller tuning.

### 7.2 Setpoint tracking performance

**Fig. 10:**
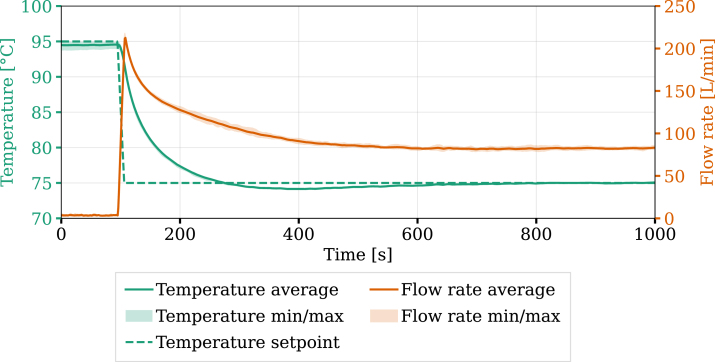
System response to a setpoint change in temperature.

A setpoint change experiment was performed to demonstrate the ability of the system to regulate the temperature using the PID control algorithm. This experiment was repeated four times under identical conditions using the following Proportional, Integral, and Derivative parameters: P=10.46, I=0.11, D=0. These values were obtained using the direct synthesis tuning method [26]. The target temperature was decreased from 95 to 75 °C. Fig. 10 illustrates both the temperature response and the corresponding change in flow rate following the setpoint change. Results include the average and the range between the minimum and maximum measured values.

After an initial peak in flow rate, the system stabilizes around 80 L min^−1^ as the system reaches steady-state. The target temperature is achieved, with negligible variations in both flow rate and temperature across experimental runs, demonstrating high repeatability of the setup. The results confirm that the hardware can effectively regulate temperature, with response characteristics indicating potential for applications requiring controlled heat extraction over time and space. These experiments demonstrate the unit’s ability to adapt to setpoint changes by actuating on the mass flow to ensure thermal management.

### 7.3 Disturbance rejection performance with a localized heat load

**Fig. 11:**
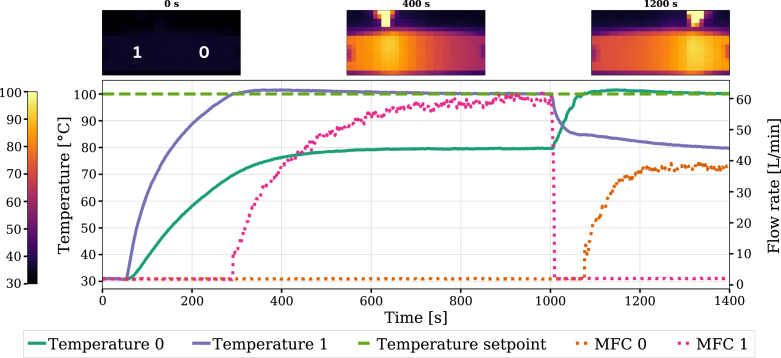
System response of two PI-controlled inlets to a localized heat-gun disturbance. The heat gun is applied over zone 1, then repositioned to be over zone 0 at 1000 s.

Disturbance rejection was evaluated with two independent PI controllers, each regulating the surface temperature over a 3 × 3 pixel region centered above its respective jet inlet. The jet arrangement from left to right was: outlet, inlet (MFC 1), closed, inlet (MFC 0), outlet. For this experiment both controllers used the following parameters: P=10, I=0.1, and D=0. The temperature setpoint under heat load was 100 °C and the experiment timeline is shown in Fig. 11.

The system was initially undisturbed. At 50s, a heat gun was positioned above region 1, causing the local temperature to rise. MFC 1 responds once the measured temperature in its control region exceeds its setpoint. By 1000 s, the system reached a new steady state. The heat gun was then moved from zone 1 to zone 0, resulting in a decrease in the flow rate of MFC 1 as its region cooled, while MFC 0 increased its flow rate to counter the new heat load.

This simple experiment demonstrates that the disturbance location can be shifted during operation and that each PI controller acts locally to regulate the temperature directly above its corresponding inlet. Notably, the two zones have different temperatures, never reaching the setpoint simultaneously. However, the device enables further characterization of the system and tuning of the control strategy.

### 7.4 Conclusion

This work successfully demonstrates a simple, low-cost, modular, and versatile experimental setup to safely test spatio-temporal thermal management strategies using technology developed by Lamarre and Raymond [9]. The proposed design and the data associated with the present work include:

1. CAD drawings with associated part numbers of components, allowing for an easy reproduction of the setup. The files are in STEP format, which facilitates scaling of parts and machining, when necessary. Replacing parts of the design assembly is also feasible.
2. PCB design files, including Gerber files with the entire PCB layout. This greatly simplifies the reproduction of the experimental setup by eliminating the need for a deeper understanding of PCB layout design. It also allows for extension of the setup, since connections with different parts and module components can be easily replaced.
3. A user-friendly open-source software to control the device. The software is written in Python using the PySide6 library and the object-oriented programming paradigm. The framework results in a highly readable and adaptable source code without sacrificing performance.
4. Custom firmwares to control DAC7578SRGER, TLA2528IRTER, and DRV8806 chips using a Raspberry Pi. Firmwares are implemented as independent Python modules, allowing for their independent use in other builds.
5. Examples of use of the setup for system characterization using step response, single PI controller performance tracking with setpoint change, and disturbance rejection performance for interacting PI controllers. The reproducibility of results highlights the robustness of the setup. For a similar setup, the benchmark results can be used as a basis of comparison for more advanced control strategies (Model-Predictive control, for instance). The proposed characterization protocols have been adopted by Vaillant et al. [25] to validate a surrogate model for this system.

The low-cost and modular nature of the setup allows users to leverage existing components to build their own application in order to interface with sub-components of the experimental setup, such as the MFCs and the solenoid valves. The openly available database [24] simplifies extending the application to testing varied control strategies and enables adaptation of the design to similar thermal management devices.

**Table A.1:** 

Designator	Component	Number	Cost per unit - USD	Total Cost - USD	Source of materials (store name)	Material type
C74	Capacitor ALUM 100UF 20% 35V SMD	2	$ 1.05	$ 2.10	DigiKey Canada	N/A
C75, C76, C77	Capacitor ALUM 100UF 20% 50V SMD	1	$ 0.43	$ 0.43	DigiKey Canada	N/A
C56, C57	Capacitor ALUM 470UF 20% 50V RADIAL TH	1	$ 1.66	$ 1.66	DigiKey Canada	N/A
C31, C33, C35, C37, C47, C49, C55, C71, C72	Capacitor CER 0.1UF 50V X7R 0402	3	$ 0.05	$ 0.16	DigiKey Canada	N/A
C1, C2, C5, C6, C7, C8, C9, C10, C12, C13, C16,	Capacitor CER 10000PF 50V X7R 0402	1	$ 0.15	$ 0.15	DigiKey Canada	N/A
C52, C53, C54, C59, C62	Capacitor CER 10UF 50V X5R 1206	8	$ 0.11	$ 0.84	DigiKey Canada	N/A
C3, C4, C11, C14, C15, C22, C30, C32, C70, C73	Capacitor CER 1UF 25V X5R 0402	26	$ 0.07	$ 1.82	DigiKey Canada	N/A
C58, C60, C61, C67, C68, C69, C78	Capacitor CER 22UF 25V X5R 1210	1	$ 0.46	$ 0.46	DigiKey Canada	N/A
C27, C28, C29, C66	Capacitor CER 4.7UF 25V X5R 0805	1	$ 0.08	$ 0.08	DigiKey Canada	N/A
C63	Capacitor CER 4700PF 50V X7R 0603	1	$ 0.05	$ 0.05	DigiKey Canada	N/A
C64	Capacitor CER 680PF 50V X7R 0402	10	$ 0.04	$ 0.35	DigiKey Canada	N/A
2233K19	Clamping Nut for 1-5/8” Strut Channel	8	$ 3.75	$ 30.00	McMaster Carr	Plastic
8560K914	clear Scratch- and UV-Resistant Cast Acrylic Sheet, 12“ x 12” x 2”	1	$ 147.68	$ 147.68	McMaster Carr	Rubber
J2, J3, J8, J9, J16, J17, J18, J19, J20, J21	Click-mate 1.5 DRVT SMT AU0.1 ETP	1	$ 2.87	$ 2.87	DigiKey Canada	N/A
	Connector 24-28AWG CRIMP TIN	3	$ 0.08	$ 0.23	DigiKey Canada	N/A
	Connector HEADER R/A 4POS 4.2MM	3	$ 1.73	$ 5.19	DigiKey Canada	N/A
J4, J5, J6, J7, J10, J11, J12, J13, J14, J15	Connector HEADER VERT 2POS	2	$ 1.16	$ 2.32	DigiKey Canada	N/A
	Connector Plug HSG 10POS 1.50MM	2	$ 0.42	$ 0.83	DigiKey Canada	N/A
	Connector Plug HSG 4POS 1.50MM	1	$ 0.41	$ 0.41	DigiKey Canada	N/A
J1, J3, J4	Connector RCPT HSG 2POS 3.00MM	1	$ 0.51	$ 0.51	DigiKey Canada	N/A
	Connector RCPT HSG 4POS 4.20MM	2	$ 0.55	$ 1.10	DigiKey Canada	N/A
J22	Connector RECPT R/A SMC 4 POS	1	$ 1.60	$ 1.60	DigiKey Canada	N/A
	Connector Socket 16AWG CRIMP TIN	2	$ 0.21	$ 0.42	DigiKey Canada	N/A
	Connector Socket 20-24AWG CRIMP TIN	1	$ 0.08	$ 0.08	DigiKey Canada	N/A
U16	DC/DC Converter 3.3-16.5V 100 W	2	$ 35.59	$ 71.18	DigiKey Canada	N/A
D22, D23, D24, D25, D27, D28, D29, D30, D32, D33	Diode GEN PURP 50V 1A SMA	1	$ 0.26	$ 0.26	DigiKey Canada	N/A
D35	Diode SCHOTTKY 30V 5A SMC	1	$ 0.56	$ 0.56	DigiKey Canada	N/A
D34	Diode ZENER 12V 500MW SOD123	1	$ 0.09	$ 0.09	DigiKey Canada	N/A
PFCQ531-04-A1C-S	Flow Controller for Air, 9-300 lpm	10	$ 1108.21	$ 11,082.10	SMC	N/A
F1, F2	Fuse BRD MT 375MA 125VAC 63VDC	10	$ 0.38	$ 3.75	DigiKey Canada	N/A
F3	Fuse BRD MT 5A 125VAC 63VDC 1206	1	$ 0.38	$ 0.38	DigiKey Canada	N/A
1388K454	Ground Low-Carbon Steel Sheet, 10“ x 10” x 1/8”	1	$ 57.32	$ 57.32	McMaster Carr	Plastic
3433K55	Heat Gun, Pistol-Style,	1	$ 212.30	$ 212.30	McMaster Carr	N/A
3313N774	High-Strength Steel Threaded Rod, Zinc Yellow-Chromate Plated, 1/2”-13 Thread Size, 1 Foot Long	4	$ 24.11	$ 96.44	McMaster Carr	Metal
U2, U4	Integrated Circuit Analog-Digital Converter (ADC), 12BIT SAR 16WQFN	4	$ 4.09	$ 16.36	DigiKey Canada	N/A
U12	Integrated Circuit CURRENT MONITOR 5% 24VQFN	10	$ 4.81	$ 48.10	DigiKey Canada	N/A
U7, U8	Integrated Circuit Digital-Analog Converter (DAC), 12BIT V-OUT 24 VQFN	2	$ 17.27	$ 34.54	DigiKey Canada	N/A
U14	Integrated Circuit linear regulator 5.5V 300MA SOT23-5	10	$ 0.56	$ 5.60	DigiKey Canada	N/A
U1, U3, U5	Integrated Circuit OPAMP GP 4 CIRCUIT 14TSSOP	5	$ 2.62	$ 13.10	DigiKey Canada	N/A
U15	Integrated Circuit REG LINEAR 3.3V 500MA DPAK	30	$ 2.54	$ 76.20	DigiKey Canada	N/A
U6, U17	Integrated Circuit XLTR VL BIDIR 8-VSSOP	1	$ 1.30	$ 1.30	DigiKey Canada	N/A
D36	LED green clear SMD	1	$ 0.23	$ 0.23	DigiKey Canada	N/A
D21, D26, D31	LED Red clear SMD	11	$ 0.23	$ 2.53	DigiKey Canada	N/A
Q2	MOSFET N-CH 30V 2.2A SUPERSOT3	1	$ 0.96	$ 0.96	DigiKey Canada	N/A
Q1	MOSFET P-CH 40V 90A TO252-3	1	$ 4.00	$ 4.00	DigiKey Canada	N/A
8983K361	Multipurpose 304 Stainless Steel Sheet	1	$ 67.38	$ 67.38	McMaster Carr	Metal
8975K87	Multipurpose 6061 Aluminum sheet, 1/2”	1	$ 11.51	$ 11.51	McMaster Carr	Metal
9246K13	Multipurpose 6061 Aluminum sheet, 3/4”	1	$ 43.89	$ 43.89	McMaster Carr	Metal
9452K448	Oil-Resistant Buna-N O-Ring, 1/4 Fractional Width, Dash Number 454	1	$ 11.42	$ 11.42	McMaster Carr	Metal
5225K516	Push-To-Connect Tube Fitting For Air, Straight Adapter, 12 Mm Tube Od x 1/2 Bspp	18	$ 26.60	$ 478.80	McMaster Carr	Plastic
5225K718	Push-To-Connect Tube Fitting For Air, Straight Adapter, For 12 Mm Tube Od x 3/8	13	$ 18.49	$ 240.37	McMaster Carr	Plastic
SC1113	Raspberry Pi 5, 16 GB, 2.4 GHz 64-bit Quad Core ARM Processor, Dual-Band 802.11ac Wi-Fi, Bluetooth 5.0	1	$ 174.53	$ 174.53	DigiKey Canada	N/A
R29, R30, R37, R38, R57, R61, R62, R63	Resistor 0 Ohm Jumper 1/16 W 0402	3	$ 0.01	$ 0.03	DigiKey Canada	N/A
R1, R3, R5, R8, R10, R12, R14, R16, R19, R21, R5	Resistor 100 Ohm 1% 1/16 W 0402	1	$ 0.01	$ 0.01	DigiKey Canada	N/A
R18	Resistor 100K Ohm 1% 1/16 W 0402	1	$ 0.01	$ 0.01	DigiKey Canada	N/A
R48	Resistor 100K Ohm 1% 1/16 W 0402	1	$ 0.04	$ 0.04	DigiKey Canada	N/A
R51	Resistor 1M Ohm 1% 1/16 W 0402	1	$ 0.01	$ 0.01	DigiKey Canada	N/A
R52	Resistor 330 Ohm 1% 1/16 W 0402	7	$ 0.01	$ 0.08	DigiKey Canada	N/A
R7	Resistor 33K Ohm 1% 1/16 W 0402	10	$ 0.01	$ 0.11	DigiKey Canada	N/A
R23, R24, R27, R28, R31, R32, R33, R34, R35, R36	Resistor 470 Ohm 1% 1/16 W 0402	2	$ 0.01	$ 0.02	DigiKey Canada	N/A
R53	Resistor 56K Ohm 1% 1/16 W 0402	1	$ 0.01	$ 0.01	DigiKey Canada	N/A
R39, R40, R41	Resistor 6.8K Ohm 1% 1/16 W 0402	5	$ 0.01	$ 0.06	DigiKey Canada	N/A
R46	Resistor SMD 0 Ohm Jumper 1/2 W 1206	3	$ 0.71	$ 2.13	DigiKey Canada	N/A
R2, R4, R6, R9, R11, R13, R15, R17, R20, R22, R2	Resistor SMD 10K Ohm 1% 1/16 W 0402	1	$ 0.02	$ 0.02	DigiKey Canada	N/A
R45, R49	Resistor SMD 20K Ohm 1% 1/5 W 0402	20	$ 0.09	$ 1.74	DigiKey Canada	N/A
R56	Resistor SMD 7.87K Ohm 1% 1/10 W 0402	9	$ 0.04	$ 0.39	DigiKey Canada	N/A
98150A170	Slide-Adjust Push-Button Nut, Hex, 1/2”-13 Thread Size	4	$ 17.14	$ 68.56	McMaster Carr	Plastic
98150A770	Slide-Adjust Push-Button Nut, Knurled-Head, 1/2”-13 Thread Size	8	$ 17.14	$ 137.12	McMaster Carr	Metal
1023N279	Straight-Flow Rectangular Manifold, Anodized Aluminum, Standard, 10 Outlets, 1/2 Npt x 3/8 Npt	1	$ 63.19	$ 63.19	McMaster Carr	Metal
33125T601	Strut Channel Bracket, 90 Degree Angle, Zinc-Plated Steel, 4“ x 4” Long	4	$ 15.24	$ 60.96	McMaster Carr	Metal
3259T31	Strut Channel Nut, Zinc-Plated Steel, 1/2“-13 Thread Size, 3/8” High	4	$ 8.77	$ 35.08	McMaster Carr	Metal
3310T517	Strut Channel, Low-Profile, Slotted Hole, Galvanized Steel	4	$ 9.38	$ 37.52	McMaster Carr	Metal
2545T81	Super-Conductive 101 Copper, Softened Temper Sheet, 12“ x 12”, 1/8” Thick	1	$ 120.92	$ 120.92	McMaster Carr	Metal
D37	TVS Diode 24VWM 38.9VC SMA	1	$ 0.47	$ 0.47	DigiKey Canada	N/A
D1, D2, D3, D4, D5, D6, D7, D8, D9, D10, D11, D1	TVS Diode 5VWM 9.2VC DO219AB	1	$ 0.22	$ 0.22	DigiKey Canada	N/A
D38	TVS Diode 80VWM SOT363	10	$ 0.17	$ 1.66	DigiKey Canada	N/A
51305K429	Universal-Thread Push-To-Connect Tube Fitting For Air And Water, Wye, 12 Mm Tube Od x 1/2 Male Pipe	9	$ 11.53	$ 103.77	McMaster Carr	Metal
N/A	Viper VP4000 Mini 500GB M.2 2230 PCIe Gen4 x4 SSD - Solid State Drive - VP4000M500GM23	1	$ 60.99	$ 60.99	DigiKey Canada	N/A

## CRediT authorship contribution statement

**Victor Oliveira Ferreira:** Writing – review & editing, Writing – original draft, Software, Methodology, Investigation, Formal analysis, Conceptualization. **Wiebke Mainville:** Writing – review & editing, Writing – original draft, Validation, Investigation, Formal analysis, Conceptualization. **Vincent Raymond:** Resources, Methodology, Investigation, Funding acquisition, Conceptualization. **Jean-Michel Lamarre:** Investigation, Funding acquisition, Conceptualization. **Antoine Hamel:** Software, Methodology, Investigation, Conceptualization. **Mikael Vaillant:** Investigation, Formal analysis, Conceptualization. **Moncef Chioua:** Supervision, Methodology, Investigation, Formal analysis, Conceptualization. **Bruno Blais:** Writing – review & editing, Writing – original draft, Supervision, Project administration, Methodology, Investigation, Funding acquisition, Formal analysis, Conceptualization.

## Appendix Bill of materials

See Table A.1.
